# A New Algorithm to Diagnose Atrial Ectopic Origin from Multi Lead ECG Systems - Insights from 3D Virtual Human Atria and Torso

**DOI:** 10.1371/journal.pcbi.1004026

**Published:** 2015-01-22

**Authors:** Erick A. Perez Alday, Michael A. Colman, Philip Langley, Timothy D. Butters, Jonathan Higham, Antony J. Workman, Jules C. Hancox, Henggui Zhang

**Affiliations:** 1 Biological Physics Group, Department of Physics and Astronomy, University of Manchester, Manchester, United Kingdom; 2 School of Engineering, University of Hull, Hull, United Kingdom; 3 Institute of Cardiovascular and Medical Sciences, University of Glasgow, Glasgow, United Kingdom; 4 School of Physiology and Pharmacology, and Cardiovascular Research Laboratories, School of Medical Sciences, University of Bristol, Bristol, United Kingdom; 5 School of Computer Science and Technology, Harbin Institute of Technology, Harbin, China; Gent University, BELGIUM

## Abstract

Rapid atrial arrhythmias such as atrial fibrillation (AF) predispose to ventricular arrhythmias, sudden cardiac death and stroke. Identifying the origin of atrial ectopic activity from the electrocardiogram (ECG) can help to diagnose the early onset of AF in a cost-effective manner. The complex and rapid atrial electrical activity during AF makes it difficult to obtain detailed information on atrial activation using the standard 12-lead ECG alone. Compared to conventional 12-lead ECG, more detailed ECG lead configurations may provide further information about spatio-temporal dynamics of the body surface potential (BSP) during atrial excitation. We apply a recently developed 3D human atrial model to simulate electrical activity during normal sinus rhythm and ectopic pacing. The atrial model is placed into a newly developed torso model which considers the presence of the lungs, liver and spinal cord. A boundary element method is used to compute the BSP resulting from atrial excitation. Elements of the torso mesh corresponding to the locations of the placement of the electrodes in the standard 12-lead and a more detailed 64-lead ECG configuration were selected. The ectopic focal activity was simulated at various origins across all the different regions of the atria. Simulated BSP maps during normal atrial excitation (i.e. sinoatrial node excitation) were compared to those observed experimentally (obtained from the 64-lead ECG system), showing a strong agreement between the evolution in time of the simulated and experimental data in the P-wave morphology of the ECG and dipole evolution. An algorithm to obtain the location of the stimulus from a 64-lead ECG system was developed. The algorithm presented had a success rate of 93%, meaning that it correctly identified the origin of atrial focus in 75/80 simulations, and involved a general approach relevant to any multi-lead ECG system. This represents a significant improvement over previously developed algorithms.

## Author Summary

Ectopic activity is associated with multiple cardiac disorders and has been implicated in the initiation of self-sustaining re-entrant excitation. Identifying the presence and origin of ectopic activity may be vital in improving diagnosis and treatment of disorders such as atrial fibrillation, and has been the subject of multiple studies. The electrical activity of the heart can be non-invasively monitored through the electrocardiogram. However, the standard 12-lead electrocardiogram may not provide sufficient information to resolve the focus of ectopic activity satisfactorily and accurately; more detailed multi-lead electrocardiograms may provide more information to be able to produce an algorithm to locate the origin of ectopic activity. Using a 3D computational atria-torso model developed in our laboratory, we simulated the electrical activity of the atria under normal and different ectopic conditions. The model was first validated by comparison to experimental data, and then used to develop an algorithm to identify the location of atrial ectopic focus using a 64-lead electrocardiogram. The algorithm developed was able to identify the origin of atrial ectopic activity in 75/80 simulations, which is a significant improvement compared to previously developed algorithms. Furthermore, the study suggests that multi-lead electrocardiograms provide significant benefits over the standard 12-lead configuration.

## Introduction

Rapid atrial arrhythmias such as atrial tachycardia (AT) and atrial fibrillation (AF) can reduce cardiac output and predispose to ventricular arrhythmias and further complications, such as stroke and even sudden cardiac death [1–3]. Both AT and AF are associated with ectopic activity—rapid and irregular spontaneous excitation originating from regions of the atria other than the cardiac pacemaker, the sinoatrial node [4]. Such activity can interrupt normal sinus rhythm and mediate the development of the self-perpetuating re-entrant excitation associated with AT/AF [5], therefore implicating an important role for ectopic activity in the initiation and recurrence of both arrhythmias.

The pulmonary vein (PV) sleeves in the left atrium (LA) are usually identified as a major source of rapid ectopic activity [6–8], and catheter ablation therapy targeting the PV sleeves is commonly used as a treatment of AF [4, 7]. However, success rates for catheter ablation therapy are not entirely satisfactory (about 50% in single-procedure ablation [9]). Consequently, repeated operations may be required, resulting in significant scar tissue in the LA. Such scarring may induce further complications, such as contributing towards a reduction in cardiac output as well as providing conduction barriers which may promote the development of re-entry [10]. Furthermore, ectopic activity is not associated with the PVs alone; focal beats have been observed to originate from multiple regions of both the left and right atria [11, 12]. Hence, identifying the presence and location of ectopic activity is important for guiding ablation therapy, which may increase success rates and reduce the need for repeated operations. Moreover, identifying atrial ectopic activity and its origins may help in the diagnosis of early onset AF [13] and lead to timely treatment, inhibiting the development of persistent or chronic AF before the occurrence of permanent electrical and structural remodelling [13].

The electrocardiogram (ECG) is the most common non-invasive method of monitoring cardiac activity. The P-wave of the ECG is associated with atrial activation; irregular ectopic atrial activity may therefore be reflected as an alteration to the P-wave morphology (PWM). Multi-electrode ECG systems, such a 64-lead ECG vest [14], provide spatially detailed mapping of the body-surface potential (BSP). However, it is unclear if such further detail provides significant benefits over the standard 12-lead ECG in terms of resolving the location of ectopic atrial activity. In this study, we have used a biophysically detailed computational model of the human atria and torso to investigate the correlation between PWM of 64-lead ECGs and the location of atrial ectopic activity, in order to develop a focus-location algorithm.

## Methods

### 3D atria-torso model and simulation of BSP and multi-lead ECG

**Figure 1:**
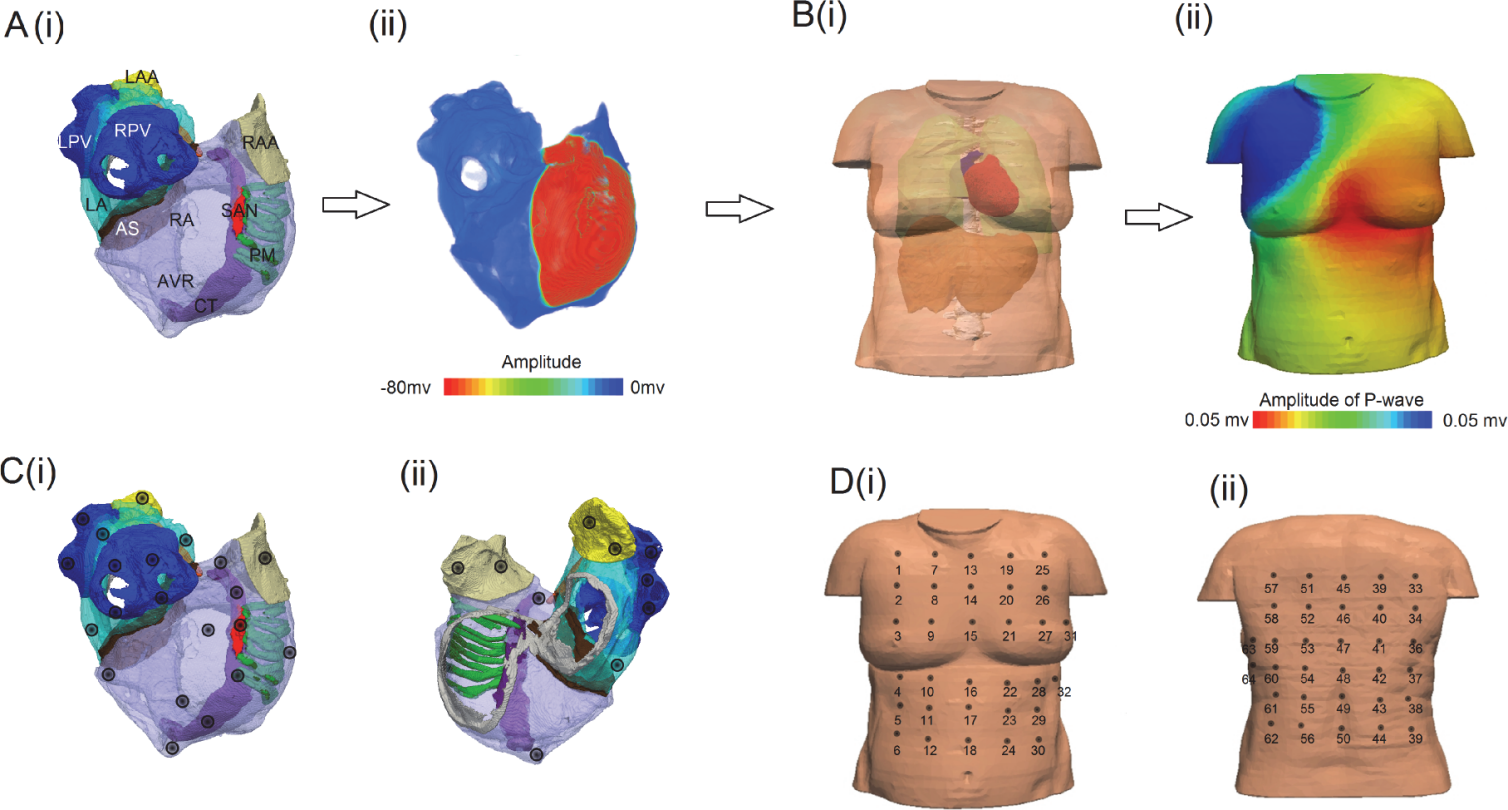
Models and procedure used to develop the algorithm. (A(i)) 3D Atrial model with the different regions of the atria included in this simulation: right atrium (RA, transparent purple), right atrial appendage (RAA, beige), pectinate muscles (PM, green), cristalterminalis (CT, solid purple), sinoatrial node (SAN, red), superior vena cava (SVC), atrio-ventricular ring (AVR, grey), right pulmonary vein (RPV, blue), Bachmann’s bundle (BB, orange), left atrium (LA, light blue), left atrial appendage (LAA, yellow), inferior vena cava (IVC) and left pulmonary veins (LPV, blue). A(ii) is a snapshot of the activation of the atria at 30ms after initiation. B(i) Torso model with all the considerations used in the simulation, we can observe the position of the atria as well. B(ii) BSP produced in our simulation, corresponding to the atrial snapshot in Aii. C(i) and (ii) indicate the different stimulated points across the surface of the atria, used for focal ectopic pacing. D Positions of the electrodes placement in the torso mesh from the front (i) and from the back (ii), for the 64-lead ECG system.

Previously we have developed a biophysically detailed computational model of the three-dimensional (3D) human atria and torso [15–17]. The model accounts for atrial anatomy [18] including segmented regions for the major anatomical structures [16] (Fig. 1Ai) and detailed atrial electrophysiology including regional differences in electrical properties [16]. The model reproduces sinus rhythm depolarisation and repolarisation patterns (Fig. 1Aii) and has been used to study the mechanisms underlying AF genesis [16, 17]. Implementation of the torso model proved useful in correlating PWM with the origin of atrial ectopic activity in a previous study [15]. However, detailed correlation between the two has not yet been established, and the torso geometry used in the previous study was idealised [15]. For a more comprehensive analysis of the relation between PWM and ectopic activity, a more realistic torso model must be used. In this study, we use our 3D model of the human atria and update the torso model in order to develop an algorithm to identify the location of focal ectopic activity in the atria (Fig. 1). Details of atrial model development and simulation protocols can be found in Colman *et al.*[16], and in S1 Text.

**Figure 2:**
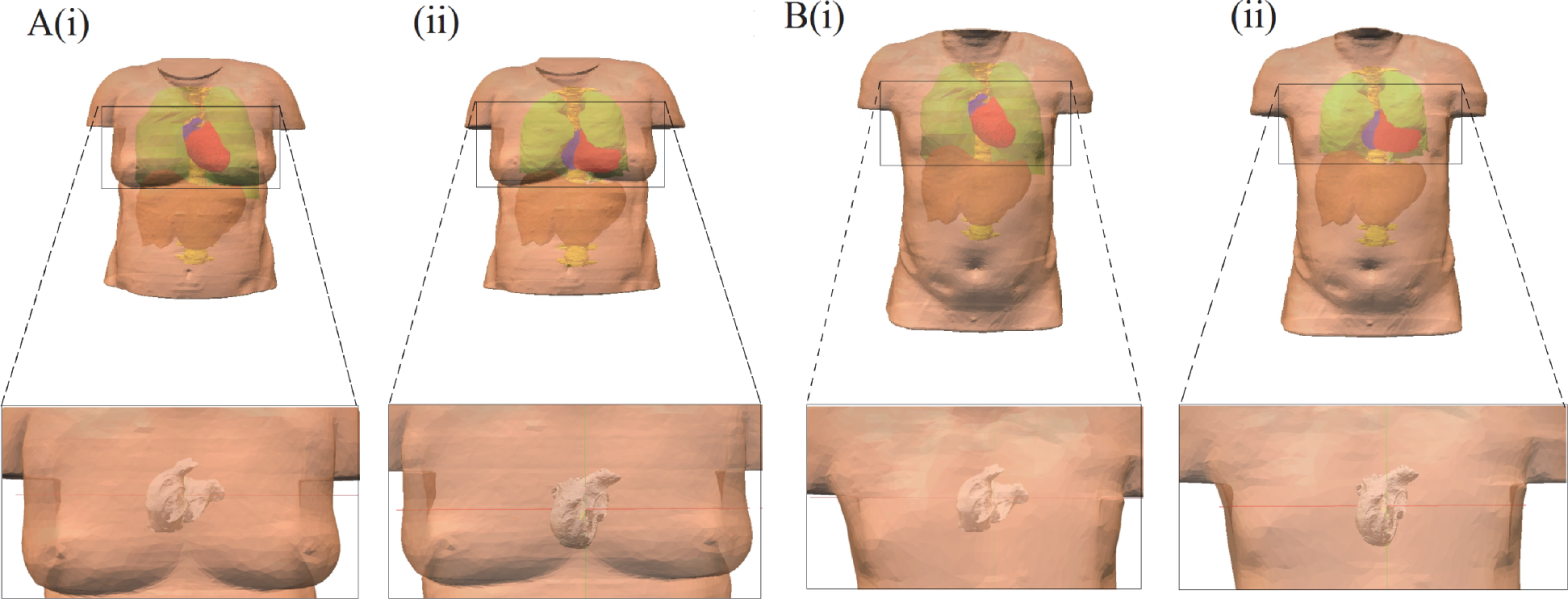
Positions of the atria inside the two different torso reconstructions used in this study. A, Female torso taken from the visible human dataset [19]. B, Male torso taken from the visible human dataset [19]. The labels (i) correspond to the position based on Ho and Sanchez-Quintana [23]. The labels (ii) correspond to the position of the atria obtained from the segmentation. The different tissues accounted for in the model are illustrated in (i) and (ii); Green-Lungs, Brown-Liver, Yellow-Spinal cord, red-Ventricles, Blue-Atria, Pink-Torso.

Two torso reconstructions are used in the present study (Fig. 2), based on segmentation of magnetic resonance imaging (MRI) images taken from the female and male visible human dataset [19], by using the software ITK-SNAP [20]. Note that the atrial model does not account for gender differences in either anatomy or electrophysiology [16] and investigation of gender differences is not the aim of this study; rather, use of multiple torso geometries ensures generality of the developed algorithm. The models account for the structure and different electrical conductivities in the lungs, liver, spinal cord and blood masses [21]. The female torso model was discretised at a spatial resolution of 0.33mm × 0.33mm × 0.33 mm[19], corresponding to that of the female atrial model [16]. Meanwhile the male torso model was discretised at a spatial resolution of 0.33mm × 0.33mm × 1 mm[19]. The 3D atrial model (Fig. 1A) [16] was then integrated into the two torso geometries and the BSP distribution was calculated through the use of a boundary element method (Fig. 1B) [22]. Two different positions of the atria inside the torso were used to account for variability between patients; one is based on Ho and Sanchez-Quintana [23] (Fig. 2 Ai, Bi), and the second one is the position of the atria obtained directly from the segmentation of the visible human female dataset (from which the atrial anatomical model was extracted) (Fig. 2 Aii, Bii). From the BSP, ECG signals can be derived by selecting elements of the torso mesh which correspond to the location of electrodes used in ECG systems. Ectopic focal activity was simulated by applying stimuli to various locations across all regions of the atria (Fig. 1C). In this study, we replicated a 64-lead ECG system which measures the BSP on the front and back of the torso (Fig. 1D) as well as the standard 12-lead ECG. All leads in the 64-lead system are unipolar: the potential at the electrode is the positive terminal and Wilson’s Central Terminal[24] is the negative terminal.

### Characterisation of the P-wave

For each lead, the P-wave was characterised by its morphology and polarity. It was indexed as positive if the amplitude of the positive peak was greater than double that of the negative peak (if there was one), and vice-versa for a negative P-wave. A biphasic P-wave is defined as one in which the second peak (positive or negative) was at least half of the amplitude of the largest peak (negative or positive). Such a definition resulted in the best performance of our focus location algorithm (described in the next section), and is not intended as a general definition for other purposes.

### Quantification of the atrial dipole evolution

The P-wave dipole pattern was constructed based on the maximum positive potentials (positive pole) and the minimum negative potentials (negative pole) in the body surface at every time step [14]. As the atrial activation evolves, the amplitude and spatial distribution of the poles across the surface of the body change dynamically. Furthermore, we constructed spatial polarity maps based on the polarity (positive/negative/biphasic) of the P-wave at each electrode location.

### Simulation of atrial focal activity

To simulate ectopic focal activity the model was stimulated by a sequence of external supra-threshold electrical pulses applied to various locations across all different regions of the atria (Fig. 1C), representing the range of ectopic foci observed experimentally [11, 12]. Stimuli were applied to each location at both slow (cycle length = 700ms) and fast (cycle length = 300ms) rates to ensure that rate dependent changes in PWM are accounted for. In each case, the P-wave resulting from the final of three stimuli was analysed.

### Focus location algorithm

**Figure 3:**
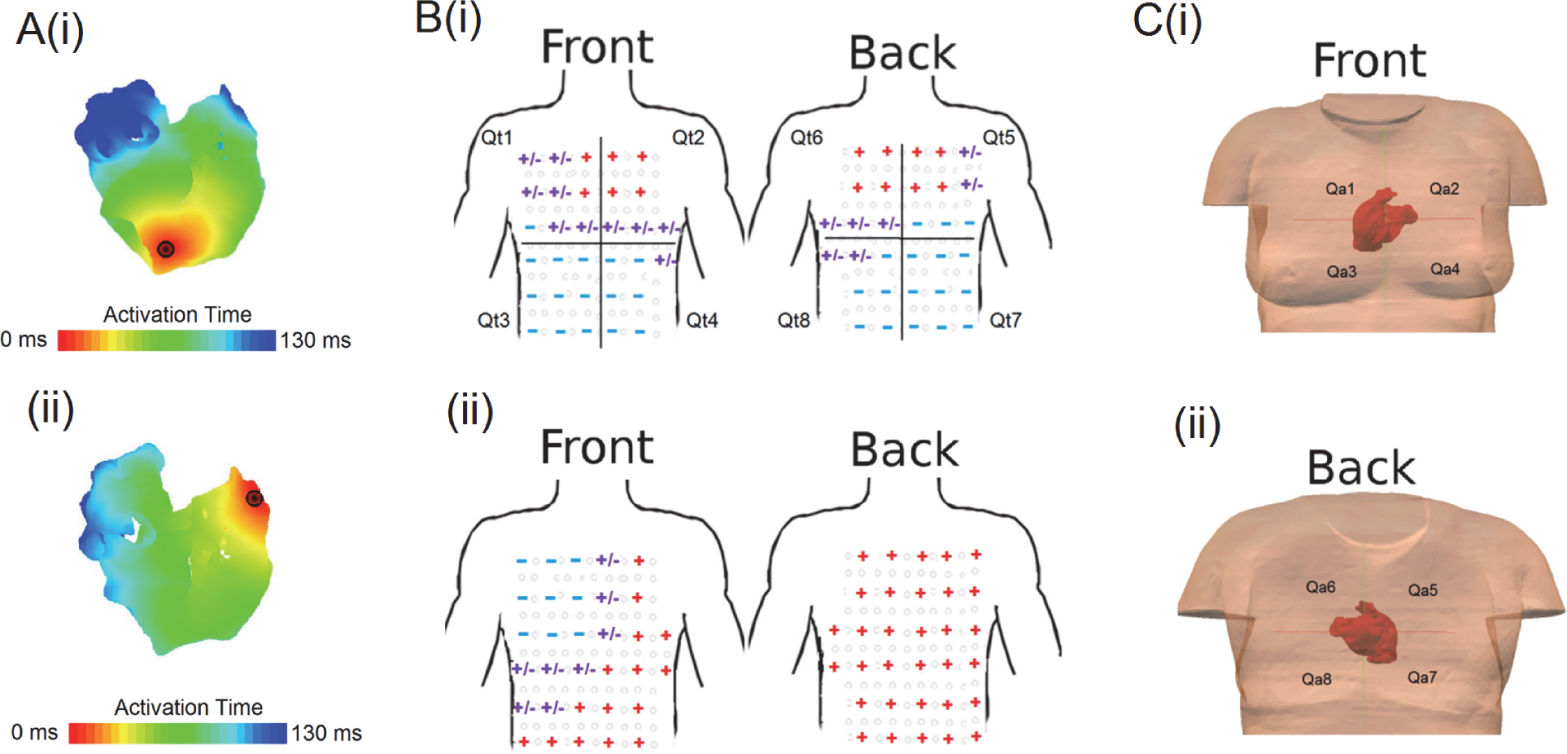
Correlation between two focal origin and the division of quadrants. Correlation between atrial focal origin (A) and the body surface polarity (B), corresponding to ectopic pacing at the inferior vena cava (Ai) right atrial appendage (Aii). In B, both the front (left panels) and back (right panels) of the torso are shown. The quadrants on the torso and the atria are illustrated in B (i),(ii) and C. Qt_i_ indexes the quadrants of the torso B(i) and (ii), and the Qa_i_ indexes quadrants of the atria C(i) and (ii).

Simulated BSP maps and ECG P-waves varied significantly with the location of the ectopic focus (Fig. 3). The P-wave polarity map offered the most effective method of quantifying such differences, offering more information than the temporal evolution of the dipole peaks while being less affected by noise than the raw P-waves. P-wave polarity maps therefore form the basis of the development of an algorithm to determine the location of an atrial focus from 64-lead ECG measurements.

To relate polarity patterns to atrial anatomical sites, both the atria and the torso were divided into two sets of quadrants, four in the anterior part and four in the posterior part of each anatomical model (Fig. 3B,C). For the torso model, Qt1 to Qt8 were used to label the quadrants (Fig. 3Bi,ii). In the atria Qa1 to Qa8 were used (Fig. 3C) where each quadrant contains corresponding anatomical regions (Table 1). Note that the position of the atria within the torso had a significant effect on PWM and the P-wave polarity map (S1 Fig.), and that the atrial anatomical locations associated with each atrial quadrant differ for both orientations considered. As such, patient variability in the orientation of the heart within the torso must be considered, and can be accounted for in this table rather than the algorithm itself, which operates by relating atrial and torso quadrants.

**Table 1:** Regions of the atria included in each quadrant for the two positions inside the torso.

**Quadrant**	**Position 1 Regions included**	**Position 2 Regions included**
Qa1	Superior-anterior part of RA, right part of RAA, superior part of the PM, superior part CT, superior part of the SAN, anterior part of the SVC	Superior-anterior part of RA, right part of RAA, SAN, PM, Superior part of CT.
Qa2	Left part of RAA	Left part of RAA
Qa3	Inferior-anterior part of the RA, inferior part of the PM, inferior anterior part of the CT, inferior part of the SAN	Inferior-anterior part of the RA, inferior anterior part of the CT, AVR, inferior-anterior part of IVC.
Qa4	inferior-anterior-left part of the RA, anterior part of the AVR	Anterior part of AVR.
Qa5	RPV, superior-right part of LA, superior part of the AS, BB, posterior part of the SVC	RSPV, superior-right part of LA, BB, SVC, superior part of AS
Qa6	LPV, superior-left part of the LA, LAA, posterior part of the AVR	LSPV, superior-left part of LA, LAA, posterior part of AVR.
Qa7	inferior part of the AS, inferior-right part of the LA, inferior-posterior part of the CT, IVC	RIPV, inferior part of AS, inferior-right part of LA, inferior-posterior part of IVC.
Qa8	inferior-left part of the LA	LIPV, inferior-left part of LA

The regions of the atria are: Right atrium (RA), right atrial appendage (RAA), pectinate muscles (PM), cristal terminalis (CT), sinoatrial node (SAN), superior vena cava (SVC), atrio-ventricular ring (AVR), right pulmonary vein (RPV), bundle branch (BB), left atrium (LA), left atrial appendage (LAA), inferior vena cava (IVC) and left pulmonary veins (LPV). Position 1 is taken from [23]. Position 2 is taken from the actual position of the atria inside its torso.

**Figure 4:**
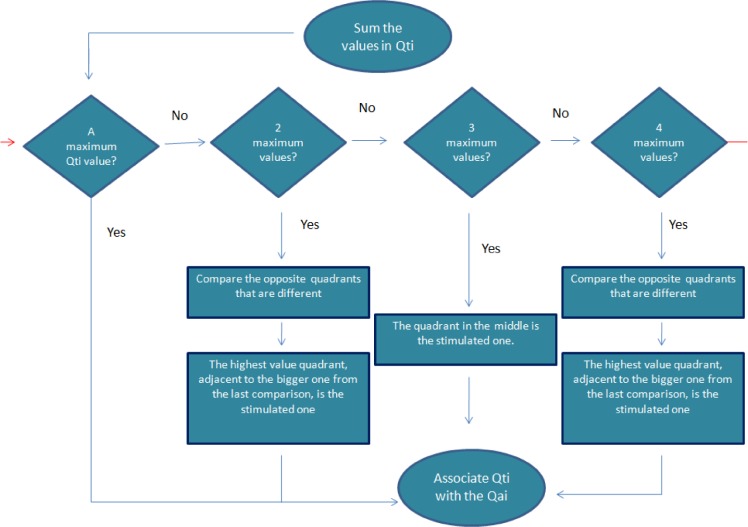
Schematic illustration of the algorithm to identify the quadrant of atrial focal origin based on 64-lead ECG P-wave values.

Schematic illustration of the algorithm is shown in Fig. 4, and details of the algorithm are described below:

1. Construct the spatial polarity map.
2. Assign a numerical value to each electrode position based on the polarity of the P-wave at that position; 2 for a negative P-wave, 1 for a bi-phasic P-wave and 0 for a positive P-wave.
3. Take the mean average of all the values in each torso quadrant, denoted Sp.
4. Determine the largest value of Sp across all quadrants, denoted Sp_max. If there is a single quadrant which contains this value (Qtx, x = 1–8), then the location of the atrial focus is in the corresponding atrial quadrant (Qax).
5. If there are multiple quadrants which contain Sp_max, then further analysis is required:
  - If the value of Sp in two quadrants is equal to Sp_max, then two adjacent quadrants must be compared. Then, the quadrant Sp_max, adjacent to the larger Sp from the second comparison, will be identified as the origin.
    Note: for example, if both superior-right and superior-left anterior quadrants have the same Sp_max value, then the Sp in the inferior-left and inferior-right anterior quadrants are compared, as long as they are different. If the inferior left has a greater Sp, then the atrial focal is in the superior-left region.
  - If there are 3 quadrants with the same Sp_max value, then the corner quadrant will be identified as the atrial focal origin.
    Note: for example, if the anterior superior-right, the anterior superior-left and the anterior inferior-left quadrants have the same maximal value, then the anterior superior-left quadrant will be the origin.
  - If 4 or more quadrants have the same Sp_max, the adjacent quadrants with different Sp will be compared, and the quadrant with a larger Sp will be identified as the origin.
    Note: for example, if the four anterior quadrants have the Sp_max, a subsequent maximal Sp in the posterior quadrants will be searched. If there is one, say the superior-right posterior one, then the superior-right anterior quadrant will be identified as the origin.

## Results

### Validation of the simulated 64-lead ECG system

**Figure 5:**
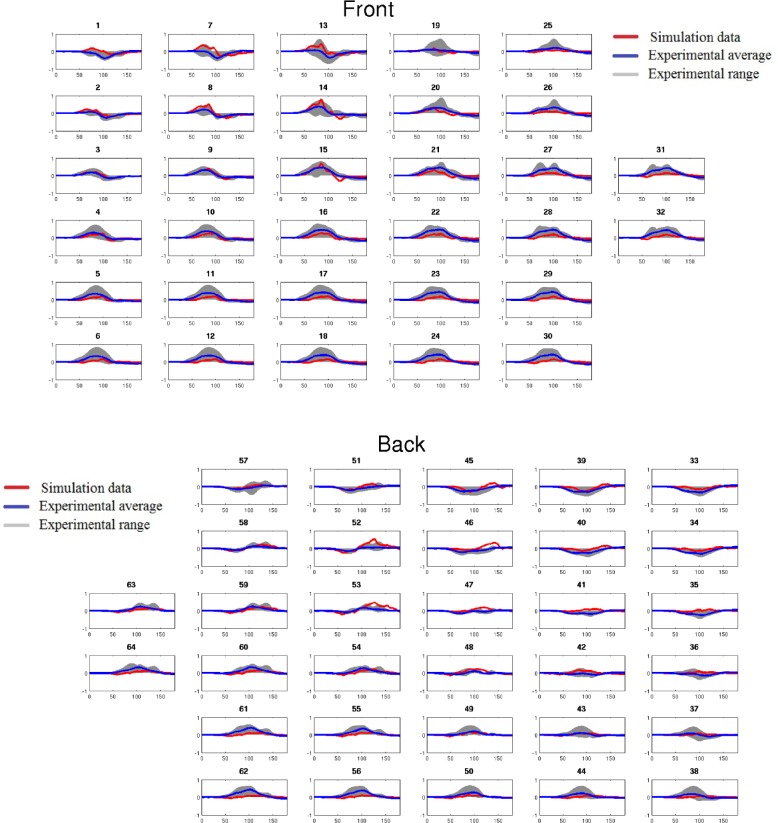
P-waves obtained from experimental data (blue line and grey shadow) and simulated (red line) data. The experimental average is the average data of 8 healthy people (blue line), and the experimental range corresponds to the maximum and minimum values of these signals (grey shadow). This measurements used the same protocol as described in [14]. Both experimental and simulated P-waves were normalized for comparison.

**Figure 6:**
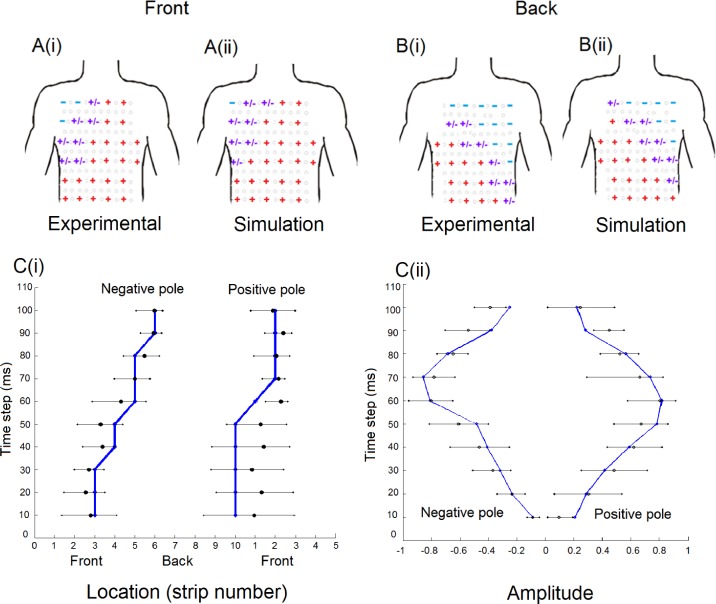
Comparison of p-waves and dipole evolution between the simulated and experimental data. A and B Comparison of the simulated 64-lead ECG P-waves polarity (ii) to experimental data (i). In this figure, the arrangement of the P-waves is set out to match electrode placement (see Fig. 1). We observed the polarity pattern of the P-waves of the experimental and simulation, in the front (A) and back (B) part of the body. The red positive sign signifies an upright P-wave, the blue negative sign represents an inverted P-wave, and the purple positive/negative sign represents a biphasic P-wave. C Spatial (i) and amplitude (ii) temporal evolution of the dipole. The black dots and lines are the experimental data and error bar taken from [14], and the blue lines and dots are obtained from our simulation during a stimuli applied to the superior part of the sino-atrial node region. In (i) the horizontal axis is a continuous scale from the first vertical line electrodes (1–6) to the last line of electrodes (33–38), without taking in to account 31, 32, 63 and 64.

Validation of the atrial activation sequence during control conditions has been discussed in [16, 17]. In order to validate the 3D atria-torso model, we first compared the simulated BSP pattern and 12- and 64- lead ECG P-waves for the control case to experimental data obtained from eight healthy subjects. It was demonstrated that the simulated data of the 64-lead ECG (Fig. 5) as well as the 12-lead ECG and BSP pattern are in fair agreement to the experimental data. Then, we further compared the simulated P-wave polarity to the experimental data. In both simulations and experimental data, the polarity of P-waves was mainly positive in the left-superior part of the body, negative in the right, inferior part of the body, and biphasic or flat in the intermediary locations (Fig. 6A, B).

To assess quantitatively the agreement between the polarity patterns in simulation and experiment, the polarity of the simulated P-wave at every electrode was compared with each experimental dataset. Inter-patient variability was quantified by also comparing experimental datasets to each-other. The simulation data showed a range of agreement between 87.1% and 94.5% with experimental data, comparable to the range observed within the experimental data of 81.5% and 93.7%. Furthermore, the simulated temporal evolution of the dipole location (Fig. 6Ci) and amplitude (Fig. 6Cii) agreed with experimental data [14]. Hence, the model was validated for the control condition, and suitable for investigating the correlation between ectopic atrial activity and P-wave profiles.

### Focus location algorithm results

The algorithm was developed based on results from 30 simulations with different atrial foci. It was then tested with 50 further simulations to determine its success rate (i.e. the proportion of cases in which the algorithm correctly identified the origin of atrial focus from the P-wave polarity pattern). In such blind tests, the success rate was 93%. Note that pacing rate affected PWM only to a small degree, and had no effect on the P-wave polarity map (S2 Fig.), hence ensuring the algorithm is appropriate for both fast and slow pacing rates.

There were five cases for which the origin identified by the algorithm did not match the actual excitation site. In those cases the mismatch was a result of the definition of a biphasic P-wave, when the PWM was highly irregular. These irregularities could impact the value of the average for each quadrant, leading to a mismatch in the location of the ectopic focus, mainly when the focal origin was close to the boundary between two or more quadrants.

Further refinements to the spatial resolution of the quadrants could be performed with the aim to improve the specificity of the algorithm for locating the focal origin site, by dividing each quadrant into sub-quadrants. Accordingly, the algorithm was updated as follows: if a quadrant adjacent to the quadrant with Sp_max has an Sp value close to that of the maximum quadrant (i.e. within 0.1 in this case), then the activation focus is determined to be in the sub-quadrant that is close to the boundary between the two quadrants (i.e. within the quadrant of maximum Sp value in close proximity to the neighbouring quadrant considered). Conversely, if the difference in values between the two quadrants is very large (i.e. greater than 0.1) then the focus of the activation is determined to be within the sub-quadrant that is far from the boundary of the two quadrants. Though such a spatial refinement improved the detection accuracy in terms of the spatial resolution, the success rate of detection showed a slight decrease, down to 89%. This could be due to the limitation of the 64-lead ECG to map the BSP.

## Discussion

In this study, we have developed a new algorithm for detecting the location of atrial focal activity using a 64-lead ECG system. The algorithm was developed using simulation data, which enabled us to correlate BSP patterns to atrial activation sequences more comprehensively than in an experimental setting.

### Computational models

The computational model implemented for this study was an update of our previous model of the human atria and torso [15–17]. The updated model has the following advantages compared to the previous model [15, 17]: (i) realistic torso meshes were used for male and female, rather than an idealised one as used in the previous studies [15, 17]; (ii) a greater level of detail was considered within the torso, including the spine and liver as well as blood masses and lungs; (iii) various, experimentally justified orientations of the atria [23] were considered. The developed atria-torso models were validated by their ability to simulate BSP patterns, 12- and 64-lead ECG PWM, 64-lead ECG polarity patterns and the spatio-temporal evolution of the dipole peaks, all of which matched to experimental data from eight healthy patients. Note that experimental P-waves were filtered and averaged over a time period of 1 minute—this has the effect of smoothing the signals compared to the simulated P-waves, for which averaging would have no smoothing effect due to the model being deterministic and subsequent P-waves being identical. Therefore, the presented models provide a useful platform for simulating atrial excitations and their BSP patterns in variant physiological conditions.

### Comparison to other models

Several human atria-torso models have been developed by other groups in previous studies [25–29], including the one by Krueger *et al*. [25], in which personalised atrial geometries were implemented for reproducing accurate patient specific P-waves. The model in that study considered fat and muscle tissue, which can affect the P-wave. However, due to the difficulty in segmenting both tissue types, few models include them [25, 30]. That model also considered soft tissues of the bowels, kidneys and spleen, which were absent in the present model. However, the simulated PWM from the present models were similar to those from Kruger *et al*. [25], suggesting that these soft tissues play only a small role in affecting the polarity of the P-waves, as also suggested in a previous study [30–32]. Furthermore, agreement of PWM between simulation and experiment were similar in both studies, despite Krueger *et al*. being patient-specific. Though other atria-torso models have been developed for simulating body surface potential maps and multi-lead ECGs, the focus of those studies were in finding the ideal number of electrodes to obtain more information of the atria as compared to the standard 12-lead ECG system [26, 27], or to create a database for detecting atrial fibrillation [33, 34]. To our knowledge, the present study is the first attempt to establish a detailed correlation between the polarity map of body surface potentials and origins of atrial ectopic focus.

### Focus location detection algorithm

**Comparison to previous algorithms**. Focus-location algorithms have been developed previously based on the standard 12-lead ECG system [35, 36], including the well-established Kistler *et al*. algorithm [11]. However, the 12-lead based algorithms have limited effectiveness due to the smaller number of electrodes that provided incomplete information on atrial excitations. In their study, Kistler *et al*. reported 93% focus detection accuracy. However, subsequent studies have found a lower accuracy [35, 36]. When we applied the Kistler *et al*. algorithm to simulation data of P-waves, an accuracy of 73% was achieved, which is within the 55–78% range observed in other studies [35, 36].

In this study, we presented an algorithm for identifying atrial focal origins based on simulated 64-lead ECG system. The developed algorithm showed a higher success rate on the same data than the Kistler *et al*. algorithm (93% vs 73% respectively). Our results suggest that the extra level of detail provided by 64-lead ECG compared to the 12-lead ECG system was useful in accurately locating atrial focal activity.

The developed algorithm has two key strengths compared to previous algorithms: (i) splitting the torso into two sets of quadrants means that the algorithm is not specific to an electrode array set up – any array which covers the front and back of the torso (symmetric or asymmetric) may be used, and the algorithm need not be adjusted. Similarly, relation of atrial anatomy to torso quadrants via a correlation table intrinsically accounts for patient variability, also without the need to adjust the algorithm itself; (ii) the algorithm is based on polarity patterns of the P-waves, rather than the detailed PWM. Whereas this does not provide a full level of detail as with PWM, such an approach has the following advantages: (a) inter-patient variations manifest as alterations in PWM but have a much smaller effect on P-wave polarity; (b) similarly, noise will not affect the P-wave polarity pattern but may have a significant effect on PWM, especially regarding bifidity; (c) we did not consider bifidity in our definition of polarity, therefore avoiding the limitations of algorithms which use bifidity, such as ambiguity in the definition of the magnitude of bump necessary to be considered bifid and the effect of noise on accentuating or reducing bifidity. Note that this was one of the primary limitations of the Kistler *et al*. algorithm [11], responsible for the majority of its errors.

Another possible approach for locating atrial ectopic foci is to implement an inverse solution. However, inverse solutions are computationally intensive and have several limitations as discussed in other studies [28, 37, 38].

**Potential application to the clinic**. In the current study, torso quadrants are associated with atrial anatomical locations by Table 1. For potential use of the algorithm in the clinic, a patient specific atria-torso correlation table could be constructed if necessary. Low resolution MRI image data can provide information of the orientation of the atria in the torso; typical MRI images would be sufficient to construct such patient specific table, and allow correlation between torso quadrants and atrial anatomical sites. As the algorithm itself is generic, it could be applicable to patients without a need for individual adjustment.

**Limitations**. The torso model lacks considerations of some other tissue types or organs (such as muscles, fat tissue, bowels, kidneys, spleen and skin) that may affect body surface potentials. However, the absence of those tissues does not have a big effect on the polarity of the P-waves [30, 31], which is the characteristic used in the present algorithm. For example, test simulations in which the conductivity of the torso was replaced by an average tissue conductivity accounting for muscle, fat and skin in various configurations demonstrates significant changes to P-wave amplitude but not to the polarity patterns (S3 Fig.). The developed algorithm was based on simulation data, lacking consideration of the measurement noise as seen in real data. However, the use of P-wave polarity in the detecting algorithm can minimise the influence of noise as this may affect the amplitude of P-wave signals, but have less impact on the P-wave polarity. Whereas polarity patterns may be affected by large degrees of noise, such signals would be unsuitable for use in any clinical diagnosis.

In the algorithm, eight quadrants were defined to cover the torso. The spatial resolution of the quadrant may require further refinement. For example, each quadrant can be split into eight sub-quadrants. However, finer spatial resolution of the quadrants may not help to improve the detection success rate as it decreases to 89% when eight sub-quadrants were used for each quadrant. Another potential limitation of the algorithm arises from the definition of a “biphasic” P-wave as it may lead to a miscalculation of the atrial activation site. Although the use of P-wave polarity overcomes the problems arising from the “bifid” definition as implemented in the Kistler *et al*. algorithm [11], the present algorithm requires a well-defined “biphasic wave” to optimise the performance of the algorithm. This “biphasic” definition leads to a better performance of the algorithm than the use of the Kistler’s “bifid” wave.

In the present study, we only tested the effectiveness of the algorithm for detecting atrial focal activity. Its use for detecting the organisation centre of rotor activity has not been performed. For that purpose, consideration of combined use of the present algorithm with vectorcardiograms [39], phase relationships [40] and correlation analysis [41] may be necessary, warranting further investigation. Finally, the algorithm was based on simulation data. Though it provides a theoretical basis for detecting atrial focus from multi-lead ECGs, it requires further tests on real ECG data from patients or animal models with known atrial foci. Nevertheless, a test of 50 simulated atrial focus activities, different to those used to develop the algorithm, was performed, which showed a similar success rate in both male and female torso models with varying atrial position.

### Conclusion

Using a biophysically detailed computer model of the human atria-torso, we have demonstrated a correlation between atrial focal origin and polarity pattern of the BSP. Based on such correlation, a new algorithm has been developed to identify the atrial origin from the BSP reconstructed from 64-lead ECG. This study provides a theoretical basis for non-invasively detecting atrial focal origins, which is important for designing AF ablation protocol, and demonstrates the advantages of multi-lead ECG systems over the standard 12-lead ECG in detecting the origin of focal activity.

## Supporting Information

S1 Fig 64-lead ECG of the two position of the atria. (DOCX)

S2 Fig 64-lead ECG and polarity maps of two pacing rates. (DOCX)

S3 Fig Lead 2 of the 64-lead ECG system. (DOCX)

S1 Text Key features of the models. (DOCX)
