# Supplementary material for: A New Algorithm to Diagnose Atrial Ectopic Origin from Multi Lead ECG Systems - Insights from 3D Virtual Human Atria and Torso
Source: PLoS Comput Biol. 2015 Jan 22;11(1):e1004026. doi: 10.1371/journal.pcbi.1004026 (PMC4303377; doi:10.1371/journal.pcbi.1004026)
Supplement: S3 Fig — (DOCX) [file pcbi.1004026.s003.docx]

Supplementary Material S3

**A new algorithm to diagnose atrial ectopic origin from multi lead ECG systems – insights from 3D virtual human atria and torso**

Erick A. Perez Alday^1*^, Michael A. Colman^1*^, Philip Langley^2^, Timothy D. Butters^1^, Jonathan Higham^1^, Antony J. Workman^3^, Jules C. Hancox^1 4^, Henggui Zhang^1+^

*^1^ Biological Physics Group, Department of Physics and Astronomy, University of Manchester, Manchester, United Kingdom,*

*^2^School of Engineering, University of Hull, Hull, United Kingdom,*

*^3^Institute of Cardiovascular and Medical Sciences, University of Glasgow, Glasgow, United Kingdom,*

*^4^School of Physiology, Pharmacology and Cardiovascular Research Laboratories, School of Medical Sciences, University of Bristol, Bristol, United Kingdom.*

^*^*Both authors have contributed equally to this study.*

*^+^Correspondence: henggui.zhang@manchester.ac.uk*

**
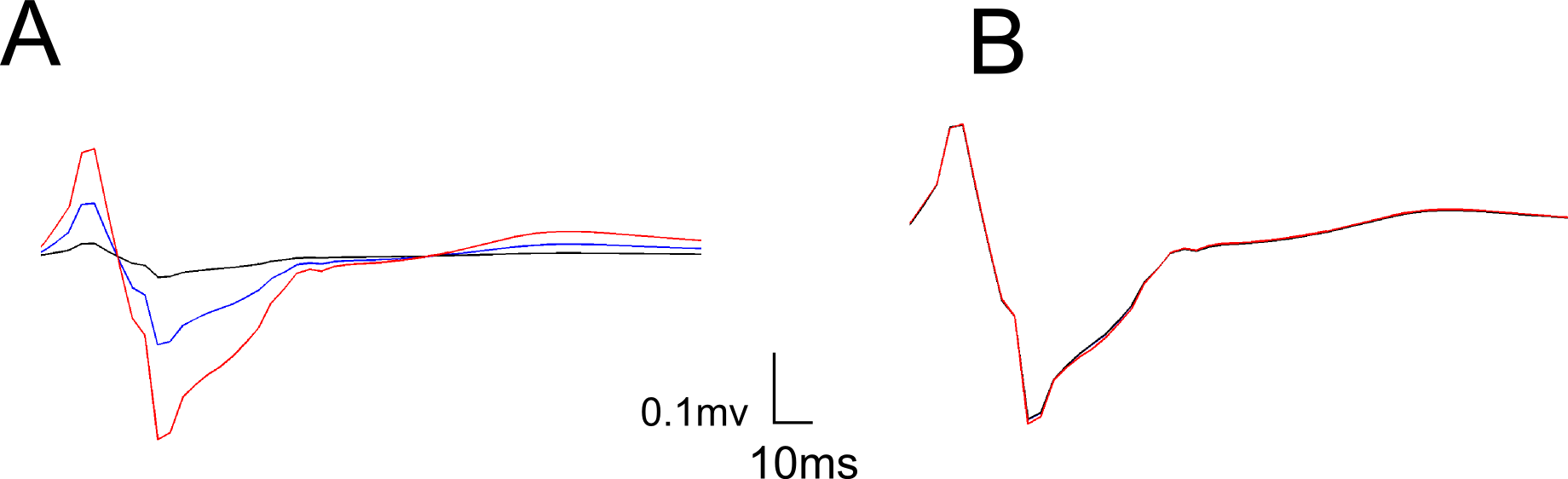
**

**Figure S3**. Lead 2 of the 64-lead ECG system. The red line in figure A is the P-wave obtained with the normal torso conductivity. Line blue is the P-wave obtained using an average tissue conductivity including muscles and fat. The black line represents the P-wave obtained using an average tissue conductivity including muscle, fat and skin. B is the same three different cases normalized. All the conductivities are taken from Grimnes and Martinsen, 2008 [21].
