# Supplementary material for: A New Algorithm to Diagnose Atrial Ectopic Origin from Multi Lead ECG Systems - Insights from 3D Virtual Human Atria and Torso
Source: PLoS Comput Biol. 2015 Jan 22;11(1):e1004026. doi: 10.1371/journal.pcbi.1004026 (PMC4303377; doi:10.1371/journal.pcbi.1004026)
Supplement: S1 Text — (DOCX) [file pcbi.1004026.s004.docx]

Supplementary Material Text S1

**A new algorithm to diagnose atrial ectopic origin from multi lead ECG systems – insights from 3D virtual human atria and torso**

Erick A. Perez Alday^1*^, Michael A. Colman^1*^, Philip Langley^2^, Timothy D. Butters^1^, Jonathan Higham^1^, Antony J. Workman^3^, Jules C. Hancox^1 4^, Henggui Zhang^1+^

*^1^ Biological Physics Group, Department of Physics and Astronomy, University of Manchester, Manchester, United Kingdom,*

*^2^School of Engineering, University of Hull, Hull, United Kingdom,*

*^3^Institute of Cardiovascular and Medical Sciences, University of Glasgow, Glasgow, United Kingdom,*

*^4^School of Physiology, Pharmacology and Cardiovascular Research Laboratories, School of Medical Sciences, University of Bristol, Bristol, United Kingdom.*

^*^*Both authors have contributed equally to this study.*

*^+^Correspondence: henggui.zhang@manchester.ac.uk*

Most of the main features of the Models and the key simulation steps has been proper referred or included in the main paper. However, we have described the key features of the models in this Supplementary Material Text S1..

**Atrial model**

The Colman et al. family[5, 16, 17] of single cell models, which accounts for the action potential in all of the major heterogeneous regions of the atria, is utilised in this study. The model is based on the Courtemanche-Ramirez-Nattel model, updated to account for recent experimental data and a compartmentalised intracellular calcium handling model [42]. Data regarding regional differences in ion current density and kinetics from both human and canine were used to derive the family of cell models, using the right-atrial cell model as a base. For further details of single cell model development and validation the reader is referred to [16].

The 3D anatomical model is extracted from the visible human female dataset and includes rule-based fibre orientation along the preferential conduction pathways of the CT, PM and BB [18]. The model was then integrated with a realistic anatomical model of the human sinus node [5, 43] and further segmented to account for the major regions of the atria, including the right and left atrial appendages, pulmonary veins, atrial-septum and atrio-ventricular ring [16].

The Forward-Euler method is used to integrate the differential equations in the single cell model. A finite-difference method is used to solve the reaction-diffusion equation describing coupling of cells in 3D. Lookup tables are also implemented to improve computational efficiency. Furthermore, initial conditions are read in dynamically based on a 1D strand model and the relevant model parameters (e.g. region, BCL) to speed convergence of the model in 3D. Such an approach allows fewer conditioning stimuli to be used in 3D, significantly improving computation time.

**Torso model**

Both 3D anatomical torso model are extracted from the visible human male and female dataset and includes the structure of the ventricles, lungs, liver, spinal cord and blood masses. The 3D atrial model was located inside the torso model with two different configurations. the first one based in the position obtained from the segmentation and the second one based on [23].

The Boundary Element Method (BEM) is used to compute the body surface potential (BSP). This method splits the body into regions with isotropic and uniform conductivity. Then, the potential is computed in the surfaces of these regions. This method is used to the Poisson’s equation

$\nabla^{2}\phi=\frac{\nabla\cdot\vec{J}_{i}}{\sigma}$, (1)

which describes the relation between the electric potential ϕ, inside a body with conductivity σ, with the active source or its applied current density $\vec{J}_{i}$. Now, using Green’s second identity [44] with the previous equation we arrive to

$\int_{V} \left( {\phi\nabla}^{2}\left( \frac{1}{r} \right)-\frac{1}{r}\frac{\left( \nabla\cdot\vec{J}_{i} \right)}{\sigma} \right)dv=\int_{S} \left( \phi\nabla\left( \frac{1}{r} \right)-\frac{1}{r}\nabla\phi\right)\cdot dS$, (2)

where$r=\left| \vec{r}^{'}-\vec{r} \right|$ is the distance from an element of the integration (dv) to an observation point inside the volume V, surrounded by a close surface S. Then, using the Dirac delta function [44], the solid angle definition [44] and assuming that the air surrounding the body has zero conductivity, we can rewrite equation (2) as

$\phi\left( \vec{r}' \right)=\frac{1}{4\pi\sigma}\int_{V} \frac{-\nabla\cdot\vec{J}_{i}}{r}dv-\frac{1}{4\pi}\int_{S} \phi\left( \vec{r} \right)d\Omega$. (3)

Then, we discretize the last equation by dividing the surface, S, in n triangles and from the definition of the solid angle, where$\Delta\Omega_{\mathrm{ii}}=-2\pi$, we can write

$\frac{\phi_{i}}{2}+\sum_{j=1j\neq i}^{n} \left( \frac{\Delta\Omega_{\mathrm{ij}}}{4\pi} \right)\phi_{i}=\frac{1}{4\pi\sigma}\int_{V} \frac{-\nabla\cdot\vec{J}_{i}}{r}dv$, (4)

Which is a system of n equations, that computes the potentials in the surface elements of the volume. Now, if we assume multiple surfaces, the generalization of the Green’s theorem for multiple surfaces can be used [44]. So, following the same proceeding, but including multiple inhomogeneities, corresponding to m surfaces, the last equation becomes

$\phi_{i}+\sum_{s=1}^{n} \left( \frac{\sigma_{s}^{-}-\sigma_{s}^{+}}{\sigma_{q}^{-}+\sigma_{q}^{+}} \right){\sum_{j=1j\neq i}^{n} \left( \frac{\Delta\Omega_{\mathrm{ij}}}{4\pi} \right)\phi}_{j}=\frac{1}{2\pi(\sigma_{q}^{-}+\sigma_{q}^{+})}\int_{V} \frac{-\nabla\cdot\vec{J}_{i}}{r}dv$, (5)

where q corresponds to the surface of the i^th^ element. Meanwhile, s is the surface of the j^th^ element. A more complete derivation of the last equation can be found in [45]. If we consider an equivalent formulation, where $\vec{J}$can be seen as a dipole density, we can define the potential in the centre of the triangles at the surface, as

$B_{i}=\frac{1}{2\pi(\sigma_{q}^{-}+\sigma_{q}^{+})}\int_{V} \frac{-\nabla\cdot\vec{J}_{i}}{r}dv$ (6)

And, if we defined a matrix **A,** depending only in the geometry of the volume conductor, we can write the set of equations (5) in a matrix form

$\mathbf{A}\phi=\mathbf{B}$**,** (7)

to compute the potential $\phi$in the surface of the n triangles. Equation (7) is used to solve the forward problem with BEM.

Supporting References

42. Koivumäki JT, Korhonen T, and Tavi P (2011) Impact of Sarcoplasmic Reticulum Calcium Release on Calcium Dynamics and Action Potential Morphology in Human Atrial Myocytes: A Computational Study. PLoS Comput Biol 7:e001067.

43. Chandler N, Aslanidi O, Buckley D, Inada S, Birchall S, et al. (2011) Computer three-dimensional anatomical reconstruction of the human sinus node and a novel paranodal area. Anat Rec Hoboken NJ 2007 294: 970–979.

44. Jackson JD (1975) Classical electrodynamics. Wiley.

45. Gulrajani RM and Mailloux GE (1983) A simulation study of the effects of torso inhomogeneities on electrocardiographic potentials, using realistic heart and torso models. Circ Res 52: 45–56.
